# Levamisole-Adulterated Cocaine-Associated Vasculopathy in a 73-Year-Old Woman: Diagnostic Challenges in Primary Care

**DOI:** 10.7759/cureus.116654

**Published:** 2026-09-21

**Authors:** Tatiana Torres Costa Lino, Astrid L Linares, Renee Peterkin-McCalman, Wayne Bryant

**Affiliations:** 1 Family Medicine, Baptist Health-University of Arkansas for Medical Sciences, North Little Rock, USA; 2 Family Medicine, University of Arkansas for Medical Sciences, Little Rock, USA; 3 Rheumatology, American College of Rheumatology, Little Rock, USA

**Keywords:** cocaine, levamisole, primary care, purpura, vasculitis

## Abstract

Levamisole, a common cocaine adulterant, can cause a distinctive skin disease combining non-inflammatory microvascular occlusion (vasculopathy) with inflammatory vessel-wall injury (vasculitis), marked by retiform purpura, skin necrosis, and atypical antineutrophil cytoplasmic antibody (ANCA) patterns. The condition is frequently misdiagnosed as primary ANCA-associated vasculitis, exposing patients to unnecessary immunosuppression.

This study aimed to describe a probable case of levamisole-associated vasculopathy in an older adult diagnosed in primary care and to highlight nonjudgmental substance-use screening in geriatric patients.

A 73-year-old woman with tobacco and cocaine use disorder presented to a family medicine clinic with progressive bilateral lower-extremity retiform purpura. She reported using cocaine by sprinkling it onto marijuana. Serologic workup showed negative antinuclear antibody, negative ANCA by indirect immunofluorescence, and negative myeloperoxidase antibodies, with an elevated proteinase-3 antibody (85 AU/mL; reference range: <19 AU/mL). Chest computed tomography showed no pulmonary involvement, and a skin biopsy was not performed. In the absence of skin biopsy, levamisole assay, or urine toxicology during the active presentation, the clinical picture, serologic pattern, and substance-use history supported a probable diagnosis of cocaine-associated vasculopathy of presumed levamisole origin rather than primary ANCA-associated vasculitis. Levamisole exposure was not confirmed by analysis of the substance used or by assay of a clinical specimen. Management included cocaine cessation counseling, topical corticosteroids, and rheumatology referral, and the lesions resolved completely within two months of cocaine cessation.

We did not find a previously published case of levamisole-associated vasculopathy diagnosed in an older adult in a primary care setting, a combination that appears to be rarely, if ever, reported. The diagnosis was probable rather than confirmed, since neither histopathology nor direct levamisole testing was available. Awareness of this presentation may reduce diagnostic error, avoid unnecessary immunosuppression, and reinforce the value of nonjudgmental substance-use assessment in older adults.

## Introduction

Levamisole is an anthelmintic and immunomodulatory agent that has become one of the most common adulterants in illicit cocaine [1,2]. By 2009, the US Drug Enforcement Administration estimated that 69% of seized cocaine contained levamisole [2]. Beyond its well-recognized hematologic toxicity (neutropenia and agranulocytosis) [1], chronic exposure can produce distinctive cutaneous disease that spans two overlapping histopathologic processes. Vasculitis refers to inflammatory injury of the vessel wall, characteristically a leukocytoclastic small-vessel vasculitis with a neutrophilic infiltrate, fibrin deposition, and nuclear debris. Vasculopathy refers to non-inflammatory occlusion of the microvasculature, characteristically thrombotic, without a primary inflammatory infiltrate. Levamisole-associated skin disease may show either process, and the two are often present together in the same specimen [3], which is why the literature commonly uses the composite term vasculopathy/vasculitis. Because histopathologic confirmation was not available in our patient, we use the broader term vasculopathy throughout this report without asserting a specific histologic mechanism. For consistency, we refer to the condition as levamisole-associated vasculopathy, by which we mean a cocaine-associated vasculopathy of presumed levamisole origin, and we use that term in preference to vasculitis wherever the process is not histologically defined. The proposed mechanism is correspondingly dual: levamisole is thought to promote antineutrophil cytoplasmic antibody (ANCA) formation and neutrophil activation, producing immune-complex-mediated leukocytoclastic injury of small dermal vessels, while a concurrent prothrombotic state, associated in some reports with antiphospholipid antibodies, produces microvascular thrombosis. Pooled biopsy series show inflammatory and thrombotic changes in broadly similar proportions and frequently in the same specimen [3], which accounts for the retiform morphology and the tendency toward cutaneous necrosis. In the largest systematic review to date, comprising 302 patients, the skin was involved in 91% of cases, with purpura in 83% and necrosis in 51%, predominantly affecting the ears and extremities [3].

The serologic signature is characteristic but frequently misleading. ANCA are detectable in roughly 89% of tested patients, most often in a perinuclear (71%) or atypical (25%) pattern, and antibodies to myeloperoxidase are found in about 73% [3]. Because this serology overlaps with primary ANCA-associated vasculitis, patients are commonly misdiagnosed and exposed to unnecessary immunosuppression [4,5].

Reported cases concentrate in middle-aged adults. Case series report mean ages of 31-43 years [6,7], and a systematic review of 192 patients reported a median age of 44 years (interquartile range: 38-50) [8]. These cases almost uniformly originate from emergency departments and from dermatology, rheumatology, and nephrology services. We describe a case identified in a primary care setting in a patient aged 65 years or older, a combination we did not find in the published cases we reviewed and that appears to be rarely, if ever, reported. This assessment is based on a targeted, non-systematic search of PubMed through June 2026, using combinations of the terms levamisole, cocaine, vasculitis, vasculopathy, purpura, and ANCA, together with the cases aggregated in two published systematic reviews [3,8]; no formal systematic review protocol was applied.

## Case presentation

History

A 73-year-old woman presented to her family medicine clinic with six months of progressive, painful purpuric lesions on both lower extremities. Associated symptoms included bilateral lower-extremity swelling and pruritus. Her medical history included osteoporosis, chronic obstructive pulmonary disease (COPD), asthma, hypertension, and depression. She had a long history of tobacco use and cocaine use disorder and reported using crack cocaine primarily by sprinkling it onto marijuana cigarettes. She had a 20-pack-year smoking history. She used crack cocaine in this manner almost daily and reported consuming approximately one alcoholic drink per week. Her medications included bupropion, valsartan, and amlodipine. She had no known drug allergies. Her family history was noncontributory. Socially, she reported significant psychosocial stress and adverse social determinants of health, and her family was unaware of her substance-use disorder.

Examination

Vital signs were notable for a temperature of 98.7°F, a blood pressure of 153/79 mmHg, a heart rate of 74 beats per minute, a respiratory rate of 18 breaths per minute, and an oxygen saturation of 91%. Skin examination revealed bilateral lower-extremity involvement with scattered retiform (stellate, net-like) purpura overlying diffuse, poorly demarcated brown-to-bronze hyperpigmented patches and plaques below the knees, with overlying excoriations and scattered crusted erosions. The remainder of the examination was unremarkable, with no synovitis and normal cardiopulmonary and abdominal findings (Figure 1 and Figure 2).

**Figure 1:**
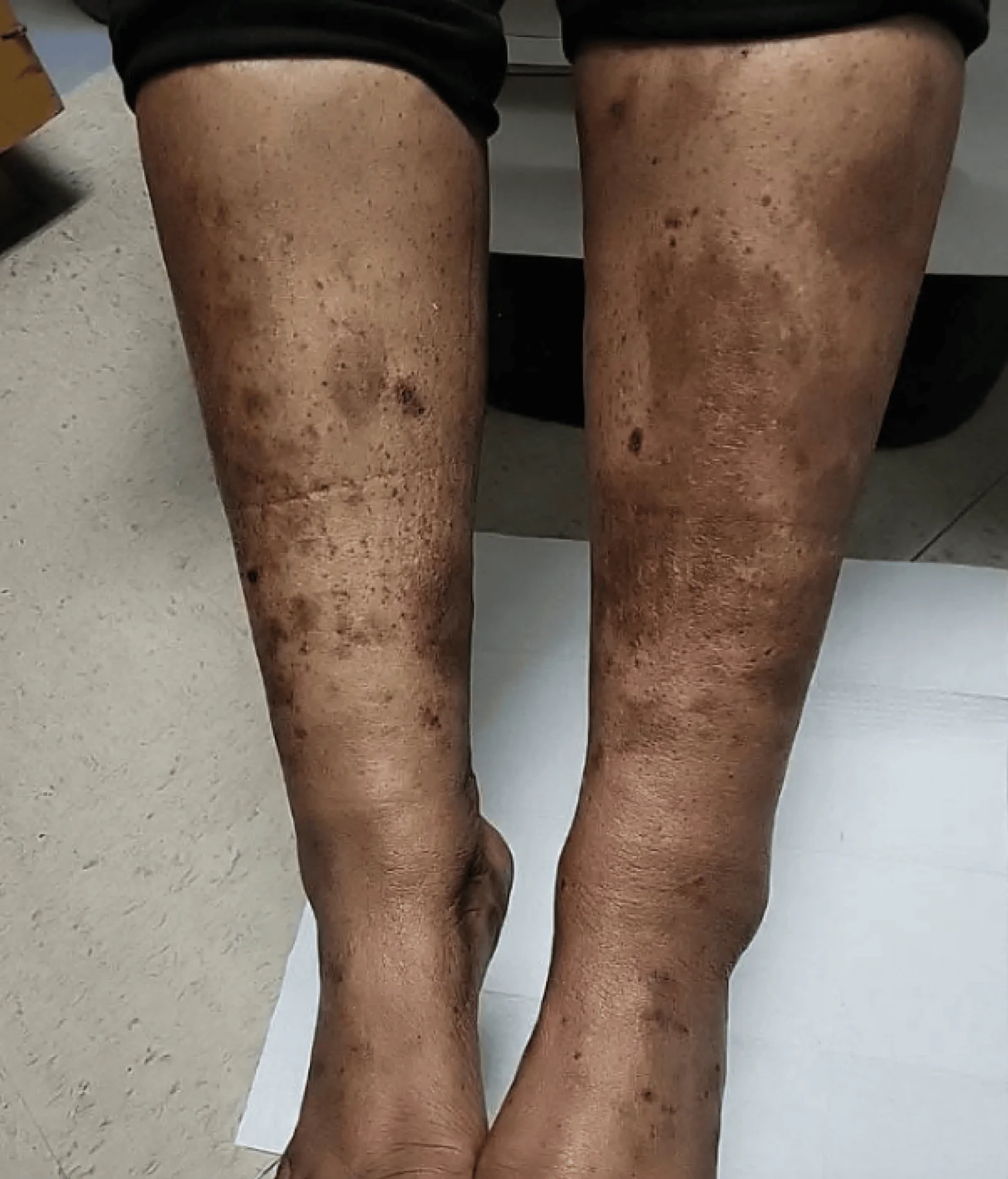
Bilateral lower extremities in a 73-year-old woman with probable levamisole-associated vasculopathy demonstrating diffuse, poorly demarcated brown-to-bronze hyperpigmented patches and plaques below the knees with scattered purpura and overlying excoriations.

**Figure 2:**
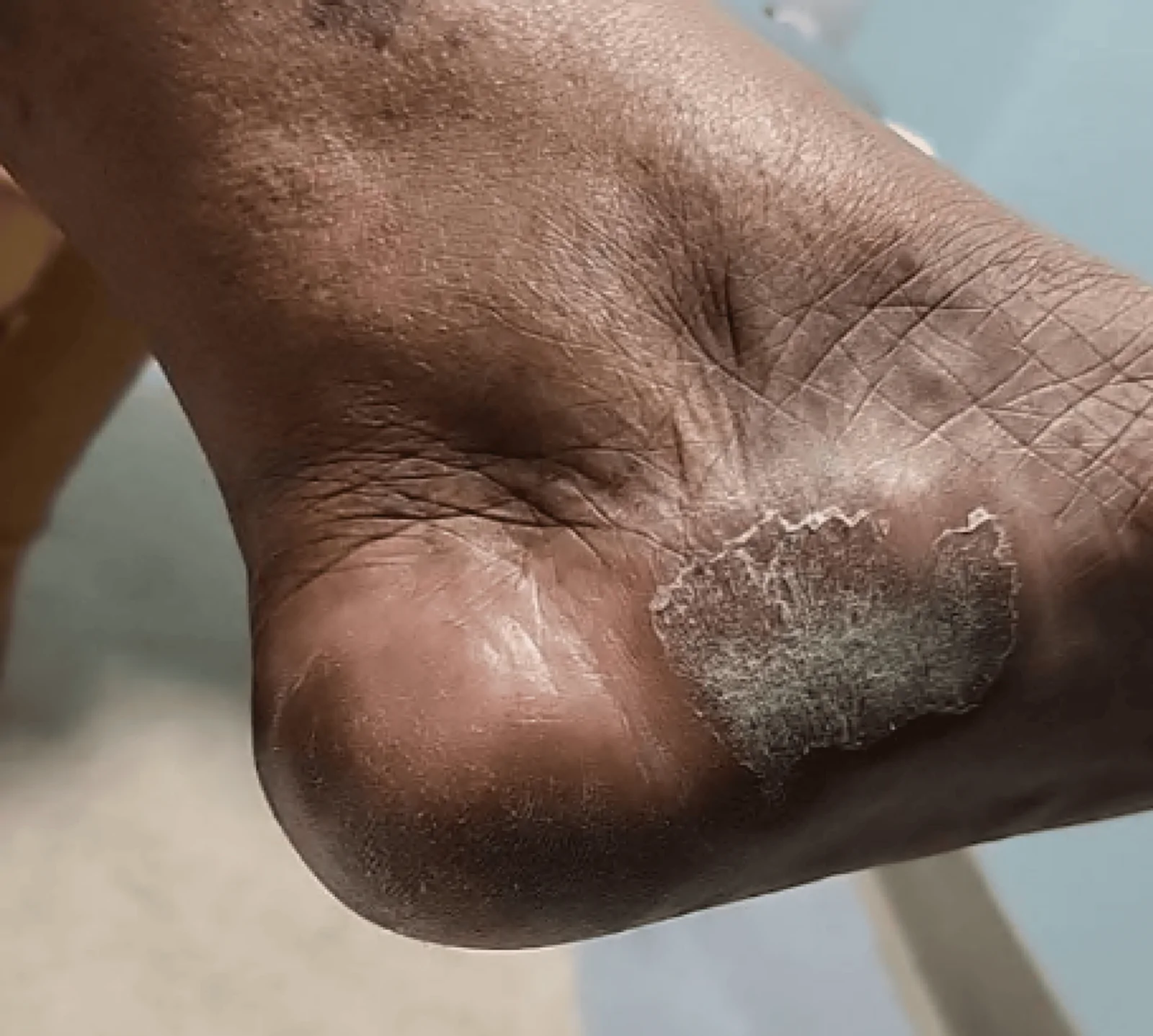
Close-up of the heel demonstrating a crusted, scaling plaque with peripheral desquamation, without frank necrosis or ulceration.

Diagnostic workup

Serologic testing showed a negative antinuclear antibody, negative ANCA by indirect immunofluorescence with no immunofluorescence pattern detected, and a negative myeloperoxidase antibody. The proteinase-3 antibody was elevated at 85 AU/mL (reference range: <19 AU/mL) by quantitative enzyme-linked immunosorbent assay (ELISA), whereas the myeloperoxidase antibody was 0 AU/mL (reference range: <19 AU/mL) by the same method. Additional testing included a negative beta-2 glycoprotein I IgA antibody, a negative lupus anticoagulant, and a negative HIV serology; renal function showed a serum creatinine of 1.0 mg/dL with an estimated glomerular filtration rate of 59.6 mL/min/1.73 m², and the urinalysis was normal. Complement levels, cryoglobulins, hepatitis B and C serologies, and inflammatory markers (erythrocyte sedimentation rate and C-reactive protein) were not obtained. A urine drug screen was not obtained at the initial evaluation; a screen performed subsequently, after cocaine cessation and clinical improvement, was negative. A complete blood count obtained at presentation showed a hemoglobin of 14.9 g/dL, hematocrit of 46.1%, and white blood cell count of 6.48×10⁹/L, without leukopenia. Computed tomography of the chest was obtained and showed no infiltrate, nodule, cavitary lesion, or evidence of alveolar hemorrhage, with findings attributable to her known COPD; there was no radiographic evidence of pulmonary involvement. A skin biopsy was not performed. Because neither histopathology, a levamisole assay, nor urine toxicology during the active presentation was available, the diagnosis was established on clinical, serologic, and exposure grounds and is best characterized as probable rather than confirmed.

Differential diagnosis

Common causes of lower-extremity purpura in an older adult were considered first. Chronic venous insufficiency with stasis dermatitis and hemosiderin deposition was the leading consideration, suggested by the bilateral distribution, the dependent edema, and the brown-to-bronze hyperpigmented patches; senile purpura, excoriation-related trauma, cellulitis, and peripheral arterial disease were also weighed. None of these accounts for the retiform, net-like morphology of the purpuric lesions superimposed on that hyperpigmentation, and none would be expected to resolve completely within two months of cocaine cessation without any change in venous, arterial, or antimicrobial management. The combination of retiform purpura and a positive proteinase-3 antibody then raised concern for primary ANCA-associated vasculitis, such as granulomatosis with polyangiitis. However, the negative ANCA by immunofluorescence and negative myeloperoxidase antibody, the absence of renal or upper-airway involvement, the absence of clinical or radiographic evidence of pulmonary vasculitis, and a substantial cocaine-use history made a drug-induced process more likely. Her oxygen saturation of 91% on room air was consistent with her known COPD and unchanged from prior clinic measurements; she reported no hemoptysis, no new dyspnea, and no pleuritic pain, and cardiopulmonary examination was normal. Taken together with the chest computed tomography findings described above, these features favored a drug-induced process [4,5]. Antiphospholipid syndrome can produce retiform purpura and was considered less likely given a negative lupus anticoagulant, a negative beta-2 glycoprotein I IgA antibody, and the absence of clinical thrombosis or pregnancy morbidity. A complete antiphospholipid antibody panel was not obtained, however: anticardiolipin IgG and IgM and beta-2 glycoprotein I IgG and IgM antibodies were not tested, and no confirmatory repeat testing at 12 weeks was performed. Antiphospholipid syndrome therefore cannot be formally excluded, although the complete resolution of the lesions after cocaine cessation, without anticoagulation, argues strongly against it. Cryoglobulinemic vasculitis typically produces palpable purpura rather than the retiform morphology observed here and is usually associated with hepatitis C; because cryoglobulins and hepatitis C serology were not obtained, this diagnosis was considered less likely on the basis of lesion morphology and clinical context rather than formally excluded, and it remains a source of residual diagnostic uncertainty. Cholesterol embolization, a relevant mimic in an older patient with vascular risk factors, characteristically follows arterial instrumentation and lacks ANCA positivity; it was not supported by the history. Other causes of retiform purpura were considered and judged unlikely on clinical grounds without dedicated testing: calciphylaxis, given normal renal function and no known disorder of calcium or phosphate metabolism; warfarin or heparin-induced skin necrosis, since she was taking neither agent; disseminated intravascular coagulation, given a normal complete blood count and the absence of bleeding or systemic illness; septic vasculitis or embolization, since she was afebrile and systemically well with a six-month indolent course; and inherited or acquired thrombophilia, for which testing beyond the lupus anticoagulant and beta-2 glycoprotein I IgA antibody was not obtained. Levamisole-adulterated cocaine vasculopathy, although not confirmed by biopsy or assay, best explained the retiform morphology, lower-extremity predominance, atypical serology, and exposure history.

Management and follow-up

Because cessation of the offending agent is the cornerstone of treatment [2,3], the patient received nonjudgmental cocaine cessation counseling. She was treated with topical triamcinolone 0.5% cream for approximately three months, with no improvement while cocaine use continued, and was referred to rheumatology for ongoing care. Systemic immunosuppression such as cyclophosphamide was deferred in the absence of organ-threatening systemic disease. After cocaine cessation, her cutaneous lesions resolved completely within approximately two months. Topical triamcinolone had already been in use for approximately three months before cessation without improvement, so the temporal association between cessation and resolution suggests that removal of the offending agent was the principal contributor. Because this was an uncontrolled single-case observation, a contribution from concurrent topical therapy cannot be excluded.

## Discussion

This case is notable for two reasons: the patient's age and the setting of diagnosis. In the published cases we reviewed, we did not find levamisole-associated vasculopathy reported in a patient aged 65 years or older nor a case described as first recognized in a primary care clinic; this combination appears to be rarely, if ever, reported. Given that published cohorts center on a median age in the mid-40s [8], a presentation at age 73 suggests the affected population may be broader than published cohorts indicate (Table 1). Beyond demographics, the case adds three features to a literature drawn largely from emergency, dermatology, rheumatology, and nephrology services. It documents the condition presenting as an indolent six-month course initially attributed to stasis-related purpura rather than as acute necrotic disease. It illustrates an atypical serologic profile, isolated proteinase-3 positivity with negative indirect immunofluorescence, that could readily be misread as granulomatosis with polyangiitis and prompt immunosuppression. And it shows a diagnosis reached, and complete resolution achieved with cessation alone, without biopsy or adulterant testing, which reflects the diagnostic resources actually available in most primary care settings.

**Table 1:** Reported levamisole-associated vasculopathy series compared with the present case. The female percentage for Muñoz-Vahos et al. [6] is derived from the reported 5:1 male-to-female ratio. In the present case, no skin biopsy was performed, and inflammatory markers, cryoglobulins, complement levels, and hepatitis B and C serologies were not obtained; the diagnosis is therefore probable rather than confirmed. Values reflect data reported in the cited sources. ANCA: antineutrophil cytoplasmic antibody; IFA: indirect immunofluorescence assay; IQR: interquartile range; NR: not reported; PR3: proteinase-3

Series (year)	Patients, N	Age, y	Female, %	ANCA positive, %	Care setting
Scoglio et al. (2026) [3]	302	NR	NR	~89	Mixed specialty
Dartevel et al. (2019) [8]	192	44 (IQR: 38-50)	63.5	93.8	Mixed specialty
Muñoz-Vahos et al. (2019) [6]	30	31 (IQR: 27-38)	~17	85	Inpatient/specialty
Gross et al. (2011) [7]	16	43 (mean)	81	93	Specialty
Present case	1	73	Female	Neg by IFA; PR3 Ab+	Primary care

The pathophysiology is dual: an immune-complex-mediated leukocytoclastic vasculitis combined with a thrombotic microangiopathy. In pooled biopsy data, vasculitis and thrombotic vasculopathy each appear in roughly 40-50% of cases, sometimes together [3,8]. This explains the retiform morphology, which reflects occlusion of dermal vasculature, and the tendency toward necrosis.

The serologic pattern is a useful diagnostic clue but not a specific one. Levamisole-associated disease characteristically produces atypical or dual ANCA reactivity; combined anti-myeloperoxidase and anti-proteinase-3 positivity was reported in 43% of patients in one review, a pattern rarely seen in primary ANCA-associated vasculitis [8]. Our patient's isolated proteinase-3 antibody with negative immunofluorescence and negative myeloperoxidase antibody is therefore less typical, since perinuclear and atypical immunofluorescence patterns and myeloperoxidase reactivity predominate among reported cases [3]. Two implications follow. First, the absence of the characteristic atypical or dual pattern does not argue against levamisole exposure, because the reported serology is heterogeneous and roughly one in 10 tested patients is ANCA-negative altogether [3]. Second, isolated proteinase-3 positivity does not by itself establish levamisole-associated vasculopathy. Proteinase-3 antibodies are the serologic hallmark of granulomatosis with polyangiitis, so this single result does not discriminate between that diagnosis and a drug-induced process. We did not identify a published levamisole-associated case reporting this exact combination of isolated proteinase-3 positivity with negative indirect immunofluorescence; our interpretation therefore rests on the broader heterogeneity of ANCA findings reported across the two systematic reviews [3,8] rather than on a specifically documented precedent. Isolated proteinase-3 positivity is also less specific for levamisole than the atypical or dual patterns described above [8]. The finding is best interpreted as one non-specific element of a composite clinical diagnosis, in which the retiform morphology, the skin-limited distribution, the absence of renal, upper-airway, and pulmonary involvement, the substance-use history, and resolution after cessation carry the diagnostic weight. Serology of this kind should prompt the consideration of a drug-induced cause rather than reflexive treatment for autoimmune vasculitis [4,5]. Attribution of the process specifically to levamisole, rather than to cocaine itself or to another adulterant, is probabilistic. It rests on two indirect lines of evidence: the reported prevalence of levamisole in seized cocaine, estimated at 69% by 2009 [2], and the concordance of this presentation with the phenotype described in pooled series [3,8]. Neither identifies the adulterant in an individual patient, and we therefore present the association as probable rather than proven. The clinical implication does not depend on resolving it, since in either case the intervention is cessation of the adulterated substance, and neither a cocaine-associated nor a levamisole-associated vasculopathy warrants reflexive immunosuppression.

The primary care context carries practical weight. With a large share of street cocaine adulterated with levamisole [1,2], the family physician may be the first and only clinician to encounter these patients. Recognition hinges on eliciting a substance-use history through nonjudgmental, routine screening, which is particularly important in older adults in whom substance use may not be suspected. Most patients improve with cessation alone: favorable outcomes were reported in 87% of patients in one systematic review [8] and clinical improvement in 93% of patients in a 30-case series [6], although relapse occurs in roughly 28% with re-exposure [8]. Accordingly, cocaine cessation is the first-line intervention. Decisions about systemic immunosuppression should be individualized according to disease severity, the presence of systemic or organ-threatening involvement, and specialist assessment, rather than driven by ANCA positivity alone. A single case of skin-limited disease without histopathologic confirmation cannot define treatment thresholds for patients with more severe or systemic presentations.

Limitations

A skin biopsy was not performed, so histopathologic confirmation of vasculitis or thrombotic vasculopathy is lacking; the diagnosis rests on the characteristic clinical morphology, serologic pattern, and exposure history. Most importantly, levamisole exposure was never confirmed. Levamisole was not assayed in a clinical specimen, confirmatory testing was not feasible owing to cost and availability, and the cocaine the patient used was not available for analysis. The role of levamisole is therefore presumed on epidemiologic and phenotypic grounds rather than established, and the case may equally be described as a cocaine-associated vasculopathy of presumed levamisole origin. A contribution from cocaine itself, or from another adulterant, cannot be excluded. The evaluation for alternative causes of retiform purpura was also incomplete. Cryoglobulins, hepatitis B and C serologies, complement levels, and inflammatory markers were not obtained; the antiphospholipid antibody panel lacked anticardiolipin and beta-2 glycoprotein I IgG and IgM testing with 12-week confirmation; and thrombophilia studies, serum protein electrophoresis, and cryofibrinogen were not performed. Cryoglobulinemic vasculitis, antiphospholipid syndrome, and the other mimics listed above were therefore judged less likely on clinical and morphologic grounds rather than formally excluded, and this residual diagnostic uncertainty should temper the attribution to levamisole. The novelty of the age and care setting rests on a targeted rather than systematic literature search, so an earlier comparable case may exist in sources we did not retrieve. Finally, this is a single case, which limits generalizability.

## Conclusions

Cocaine-associated vasculopathy of presumed levamisole origin should be considered in patients with retiform purpura and a history of cocaine use once common causes of lower-extremity purpura, such as stasis dermatitis, trauma, and infection, have been addressed, including in older adults and in primary care settings, where it has seldom been described. A positive proteinase-3 or other ANCA result does not equate to primary autoimmune vasculitis. In the present case, the diagnosis was probable rather than confirmed: neither histopathology nor direct levamisole testing was available, and the attribution to levamisole rests on clinical, serologic, and epidemiologic inference. Early recognition, nonjudgmental substance-use assessment, and cessation of the implicated substance can prevent diagnostic error and unnecessary immunosuppression. A single case cannot establish general treatment recommendations, and decisions about systemic immunosuppression should remain individualized according to disease severity, systemic involvement, and specialist assessment.
